# Effects of Late-Passage Small Umbilical Cord–Derived Fast Proliferating Cells on Tenocytes from Degenerative Rotator Cuff Tears under an Interleukin 1*β*-Induced Tendinopathic Environment

**DOI:** 10.1007/s13770-024-00673-x

**Published:** 2024-11-05

**Authors:** Ah-Young Lee, Ju-Young Park, Sam Joongwon Hwang, Kwi-Hoon Jang, Chris Hyunchul Jo

**Affiliations:** 1 Department of Orthopedic Surgery, SMG-SNU Boramae Medical Center, Seoul National University College of Medicine, Dongjak-Gu, Seoul, 07061 Korea; 2 Institute of Reproductive Medicine and Population, Medical Research Center at, Seoul National University, Jongno-Gu, Seoul, 03087 Korea; 3 Department of Translational Medicine, Seoul National University College of Medicine, Jongno-Gu, Seoul, 03080 Korea

**Keywords:** Tendinopathy, Late-passage, Mesenchymal stem cells

## Abstract

**Background:**

Tendinopathy is a chronic tendon disease. Mesenchymal stem cells (MSCs), known for their anti-inflammatory properties, may lose effectiveness with extensive culturing. Previous research introduced “small umbilical cord–derived fast proliferating cells” (smumf cells), isolated using a novel minimal cube explant method. These cells maintained their MSC characteristics through long-term culture. Thus, the purpose of the present study was to assess the anti-inflammatory effects of late-passage smumf cells at P10 on tenocytes derived from degenerative rotator cuff tears in a tendinopathic environment.

**Methods:**

The mRNA expression with respect to aging of MSCs and secretion of growth factors (GFs) by smumf cells at P10 were measured. mRNA and protein synthesis in tenocytes with respect to the tenocyte phenotype, inflammatory cytokines, and matrix- degradation enzymes were measured. The inflammatory signal pathways involving nuclear factor kappa B (NF-κB) and mitogen-activated protein kinase (MAPK) in tenocytes were also investigated. The proliferative response of degenerative tenocytes to co-culture with smumf cells over 7 days in varying IL-1*β* induced tendinopathic environments was investigated.

**Results:**

smumf cells at P10 showed no signs of aging compared to those at P3. smumf cells at P10, secreting 2,043 pg/ml of hepatocyte growth factor (HGF), showed a 1.88-fold (*p* = .002) increase in HGF secretion in a tendinopathic environment. Degenerative tenocytes co-cultured with smumf cells showed significantly increased protein expression levels of collagen type I (Col I) and the Col I/III ratio by 1.46-fold (*p* < .001) and 1.66-fold (*p* < .001), respectively. The smumf cells at P10 reduced both mRNA and protein expression levels of matrix metalloproteinases-1, -2, -3, -8, -9, and -13 in tenocytes and attenuated NF-κB (phosphorylated IκBα/IκBα and phosphorylated p65/p65) and MAPK (phosphorylated p38/p38 and phosphorylated JNK/JNK) pathways activated by IL-1*β*. Removal of IL-1*β* from the co-culture accelerated the growth of tenocytes by 1.42-fold (*p* < .001). Removal of IL-1*β* accelerated tenocyte growth in co-cultures.

**Conculsion:**

Late-passage smumf cells exert anti-inflammatory effects on tenocytes derived from degenerative rotator cuff tears under a tendinopathic environment, primarily through the secretion of growth factors (GFs).

**Supplementary Information:**

The online version contains supplementary material available at 10.1007/s13770-024-00673-x.

## Introduction

Tendinopathy is a degenerative tendon disease characterized by pain, swelling, and tenocyte dysfunction caused by chronic inflammation [1–5]. The release of proinflammatory cytokines such as tumor necrosis factor-alpha (TNF-α), interleukin (IL)-1*β*, IL-6, and IL-8 suppresses collagen synthesis and triggers vasodilation, angiogenesis, and the expression of matrix metalloproteinases (MMPs) [6]. These effects are predominantly mediated by the nuclear factor kappa B (NF-κB) and mitogen-activated protein kinase (MAPK) pathways, which are implicated in linking inflammation to joint cartilage degeneration in osteoarthritis (OA) [7, 8]. Consequently, anti-inflammatory therapy is expected to play a crucial role in future clinical treatment strategies for tendinopathy [9].

Mesenchymal stem cells (MSCs) have garnered significant attention in regenerative medicine for their potent anti-inflammatory properties, particularly in the treatment of various musculoskeletal conditions [10]. The secretomes of MSCs, comprising cytokines and growth factors (GFs), are pivotal in counteracting the catabolic and anabolic imbalances caused by local inflammation or tissue degeneration, thereby promoting tendon healing [11]. Recent studies have utilized MSCs to deliver specific GFs to targeted tissue regeneration sites, either by employing cells that naturally secrete these factors or by engineering these cells to overexpress GFs of interest [12].

In contrast to proof-of-concept laboratory studies or early-stage clinical trials, real-world clinical applications require substantial numbers of cells, making it essential to cultivate large quantities from a single tissue source [13, 14]. This approach minimizes the need for invasive procedures that can cause patient discomfort and helps avoid batch fluctuations associated with using multiple sources. Such fluctuations complicate the maintenance of consistent cell quality and significantly escalate the costs of quality control. However, generating a large number of cells from a single source necessitates rapid cell proliferation while avoiding cellular aging or DNA damage [15]. To achieve this, it is crucial to select a high-quality tissue source, develop a specialized isolation technique to extract cells with high regenerative potential, and utilize culture methods that preserve stemness.

In a previous study, we introduced a distinct MSC subtype, termed “small umbilical cord-derived fast proliferating cells” (smumf cells), isolated from the umbilical cord (UC) using an innovative minimal cube explant (MCE) isolation technique [16]. These smumf cells secreted a greater variety of bioactive factors, including basic fibroblast growth factor (bFGF), and exhibited superior proliferation ability compared to UC MSCs isolated by enzymatic digestion. In addition, smumf cells maintained stable characteristics, including proliferation, cell size, CFU-F, and resistance to senescence, over long-term culture compared to bone marrow-derived MSCs (BM MSCs) [16]. These cells also demonstrated superior tendon regeneration in a rat model of full-thickness rotator cuff tendon defects compared to BM MSCs and umbilical cord blood-derived mesenchymal stem cells (UCB MSCs) [17].

Although MSCs have shown promising therapeutic benefits, the effects of late-passage MSCs on tendinopathy have yet to be explored. Therefore, the current study aims to evaluate the anti-inflammatory capacity of late-passage smumf cells on tenocytes from degenerative rotator cuff tears under an IL-1*β*-induced tendinopathic environment. We hypothesize that late-passage smumf cells at passage 10 (P10) would exert anti-inflammatory actions through GFs on tenocytes by suppressing NF-κB and MAPK signaling pathways.

## Materials and methods

### Isolation and culture of tenocytes, smumf cells, and BM MSCs

All patients from whom tissue specimens were harvested provided informed consent. Human tendon tissues (*n* ≥ 3) were obtained from patients undergoing arthroscopic rotator cuff repair with a mean age of 64.8 ± 4.2 years. Tenocytes were isolated and cultured as previously described [18]. Tendon tissues were washed twice with Dulbecco’s phosphate-buffered saline (DPBS; Welgene, Daegu, Korea) and finely minced into 1–2 mm fragments. Cells were isolated by treating with 0.3% collagenase II (Worthington, Lakewood, NJ, USA) for 2 h in high-glucose Dulbecco’s modified Eagle’s medium (HG-DMEM; HyClone, Logan, UT, USA) containing antibiotic–antimycotic solution (100 U/ml penicillin, 100 μg/ml streptomycin, and 0.25 μg/ml amphotericin B; Welgene) with gentle agitation. After the same volume of DPBS was added, undigested tissue was removed using a 100 μm cell strainer (SPL Life Sciences, Pocheon, Korea), and cells were collected by centrifugation, washed twice, and resuspended in growth medium (HG-DMEM supplemented with 10% fetal bovine serum (FBS; HyClone) and antibiotic–antimycotic solution). Cells were plated at a density of 2–5 × 10^4^ cells/cm^2^ at 37 °C in a humidified 5% CO_2_ atmosphere. The medium was replaced every 2–3 days. When cells reached 80% confluence, they were detached by incubation for 5 min with trypsin-ethylenediaminetetraacetic acid (EDTA) (0.05% trypsin, 0.53 mM EDTA; Welgene), washed, and then replated at a ratio of 1:3. Tenocytes used for experiments did not exceed P5. smumf cells were isolated as previously described [16]. Briefly, UCs (*n* ≥ 3) were washed twice with DPBS to remove blood products. After measuring the length and weight of the total UC, cut them into 2–4 mm pieces using surgical scissors. UC pieces were aligned at regular intervals in 150 mm culture dishes (total amount: 1 g) and allowed to firmly attach to the bottom of the dish for 1 h in a 5% CO_2_ incubator with humidified air at 37 °C. The growth medium consisting of LG-DMEM supplemented with 10% FBS and an antibiotic–antimycotic solution was gently poured into the culture dishes. The culture medium was changed every 2–3 days. When cells reached 80% confluency, they were detached by incubation for 3 min with trypsin–EDTA, and the remnant tissue was filtered out using a 100 μm cell strainer. Then, cells were collected by centrifugation at 500 × g for 5 min at 20 °C and then replated at a density of 3,333 cells/cm^2^, which is P1. All experiments were conducted using smumf cells at P10 as a late-passage culture [15, 19–24]. BM MSCs were isolated as previously described [16]. Extracted BM samples (*n* ≥ 3) were diluted twice with DPBS and layered on top of Ficoll-Paque^TM^ Premium (GE Healthcare, Uppsala, Sweden) at a ratio of 1:2. This was then centrifuged at 400 g (with brake off) for 30 min at 20 °C. The uppermost layer was aspirated and discarded. The mononuclear layer was collected and diluted three times with DPBS. This was centrifuged at 400 × g for 5 min and washed again with DPBS. The supernatant was discarded, and the pellet was resuspended with 10 ml of growth medium (low glucose Dulbecco’s Modified Eagle’s Medium (LG-DMEM) containing 10% FBS and antibiotic–antimycotic solution). This was then centrifuged at 400 × g for 5 min. The cellular pellet was resuspended in a growth medium. The cells were seeded onto conventional tissue culture plates at a concentration of 1 × 10^5^ cells/cm^2^ and incubated in a 5% CO_2_ incubator with humidified air at 37 °C. The medium was replaced every 2–3 days. When cells reached 80% confluence, they were split at a ratio of 1:4. BM MSCs from P3 and P6–P10 were used in all experiments.

### Affymetrix cytoScan HD chip analysis of copy number variations (CNVs)

DNA was isolated using a Qiagen DNeasy blood and tissue kit (Qiagen, Hilden, Germany) according to the instructions provided by the manufacturer. A total of 250 ng of genomic DNA were digested with Nsp1 for 2 h at 37 °C. The digested DNA was purified and ligated with primer/adaptors at 16 °C for 3 h. Amplicons were produced by performing PCR using primers provided by the manufacturer (Affymetrix, Santa Clara, CA, USA) on the ligation products using the following program: 94 °C for 3 min, then 30 cycles of 94 °C 30 s, 60 °C for 45 s and 65 °C for 15 s. This was followed by extension at 68 °C for 7 min. The PCR products were then purified and digested for 35 min at 37 °C to fragment the amplified DNA. The fragmented DNA was then labeled with biotinylated nucleotides through terminal deoxynucleotide transferase for 4 h at 37 °C. Fragmented DNA were hybridized with a pre-equilibrated Affymetrix chip Cytoscan HD chip at 50 °C for 16 ~ 18 h. The washing and scanning procedures for CytoscanHD chips were conducted according to the manuals provided by Affymetrix, Inc. The Cel files were produced using the AGCC software from Affymetrix, Inc. (Affymetrix).

### Indirect co-culture

An indirect co-culture was established using a previously reported method with minor modifications [25]. smumf cells at P10 were seeded at a concentration of 6 × 10^4^ cells at the bottom of 24-well plates and tenocytes were seeded in 0.4 μm pore size trans-wells (Corning, Glendale, AZ, USA) at a concentration of 3 × 10^4^ cells/insert. After cultivation for 2 days, the tenocytes were changed to HG-DMEM with 2% FBS containing IL-1*β* (10 ng/ml; PeproTech, Cranbury, NJ, USA) and the cells were cultured for 6 h. And then, the transwells (tenocytes) were transferred into the 24-well plates (smumf cells) and the co-culture was maintained for 48 h in LG-DMEM with 2% FBS containing IL-1*β*. The experiment was performed in duplicate.

### Conditioned medium (CM) preparation and treatment

smumf cells at P10 were seeded at a density of 3 × 10^4^ cells/cm^2^ in growth medium. Cells grown to 80–90% confluence were washed twice with DPBS and then cultured for 24 h in serum-free medium (LG-DMEM) containing IL-1*β* (10 ng/ml). Cells were washed twice with DPBS and then incubated with fresh LG-DMEM for an additional 24 h. The smumf CM was harvested, centrifuged to remove cellular debris, and stored in 1 ml aliquots at -80 °C until further use. For protein assessment, tenocytes were pretreated with IL-1*β* at a concentration of 10 ng/ml for 6 h and treated with 50% smumf CM in the presence of IL-1*β* for 24 h after media change. To evaluate the NFκB signaling pathway, cells were treated with IL-1*β* (10 ng/ml) and 50% smumf CM for 60 min. To assess the MAPK signaling pathway, cells were treated with IL-1*β* (1 ng/ml) and 50% smumf CM for 30 min. The experiment was performed in duplicate.

### Cell viability assay

For the cell proliferation assay, tenocytes were co-cultured with or without smumf cells for 1, 2, or 7 days in normal, IL-1*β*-treated, and IL-1*β*-washed environments, respectively. After separation of trans-wells from 24-wells, EZ-Cytox cell viability assay solution WST-1 (Daeil Lab Service, Seoul, Korea) was added to each well and incubated for 2 h at 37 °C in a humidified 5% CO_2_ atmosphere. Optical densities were measured at 450 nm using the ELISA reader (Molecular Devices, San Jose, CA, USA). Single cultures of tenocytes were used as controls. The experiment was performed in triplicate.

### RNA extraction and reverse transcription quantitative polymerase chain reaction (RT-qPCR)

Total RNA was isolated with the eCube RNA Mini kit (PhileKorea Technology, Seoul, Korea) according to manufactures instructions and concentration and purity were determined by NanoDrop 2000 (Thermo Fisher Scientific, Wilmington, DE, USA). First-strand complementary DNA (cDNA) was synthesized from 1 μg total RNA with SuperioScript III cDNA Synthesis Kit (Enzynomics, Daejeon, Korea) according to manufactures instructions. RT-qPCR was performed by utilizing LightCycler®480 (Roche Applied Science, Mannhein, Germany), SYBR Prime Script™ RT-qPCR kit (Takara Biotechnology, Dalian, China), and TaqMan® Gene Expression Assay kit (Applied Biosystems, Austin, TX, USA) according to manufactures instructions. The mRNA expression levels were normalized to the housekeeping mRNA glyceraldehyde-3-phosphate dehydrogenase (GAPDH) and relative expression levels were analyzed using the 2^−ΔΔCq^ method [26]. The experiment was performed in duplicate. The RT-qPCR primers and probes are listed in supplementary Table S1 and S2.

### Human growth factor array

To analyze the GFs secreted by smumf cells at P10, IL-1*β* (10 ng/ml)-stimulated tenocytes were co-cultured with or without smumf cells for 2 days. The supernatants were harvested, centrifuged, and frozen at -80 °C until analysis. Human Growth Factor Array C1 (RayBiotech, Norcross, GA, USA) containing antibodies to detect 41 proteins was used to perform a semi-quantitative evaluation of proteins according to the manufacture instructions. Membrane signals were visualized by the LAS-4000 device (GE Healthcare) and the spot intensities were calculated by Image J with the Protein Array Analyzer macro. For comparison, each spot intensities were normalized according to the positive controls and analyzed by RayBiotech analysis tool software, which was provided by the supplier. Single cultures of tenocytes were used as controls.

### Enzyme-linked immunosorbent assay (ELISA)

To determine HGF secretion levels, tenocytes were co-cultured with or without smumf cells in the presence or absence of IL-1*β* (10 ng/ml) for 2 days. The supernatants were harvested, centrifuged, and frozen at -80 °C until analysis. The human HGF ELISA kit (R&D Systems, Minneapolis, MN, USA) was measured according to the manufacture instructions. The experiment was performed in triplicate.

### Western blot analysis

To harvest tenocytes, cells were washed with PBS and lysates buffer containing RIPA lysis and extraction buffer (Thermo Fisher Scientific) and 100X protease/phosphatase inhibitor cocktail (Cell Signaling Technology, Danvers, MA, USA) for 30 min on ice. The lysates were centrifuged for 20 min at 13,200 × g, 4 °C The protein concentration was determined using the BCA method (Thermo Fisher Scientific) and subsequently mixed with SDS sample buffer (Bio-Rad, Hercules, CA, USA), followed by boiling at 98 °C for 10 min. The proteins were separated by SDS-PAGE gels, and then transferred to PVDF membrane (Millipore, Burlington, MA, USA). Membranes were blocked with 5% non-fat skim milk or 5% Bovine Serum Albumin (Sigma Aldrich, Saint Louis, MO, USA), and then the primary antibodies were incubated overnight at 4 °C. The proteins were detected by EZ-Western Lumi Femto (Daeil Lab Service) using ChemiDoc MP Imaging System (Bio-Rad). Single cultures of tenocytes were used as controls. The antibodies used in this study are listed in supplementary Table S3.

### Statistical analysis

All data are presented as the means ± standard deviations (SD) from a representative experiment conducted in triplicate. The statistical significance of differences was determined using one-way analysis of variance (ANOVA) followed by Tukey’s post hoc test for comparisons among three groups. For comparisons between two groups, the Mann–Whitney U test was employed. All statistical analyses were performed with SPSS software version 20 (SPSS Inc., Chicago, IL, USA). Differences of *p* < 0.05 were considered statistically significant.

## Results

### Alterations in aging hallmarks in smumf cells and BM MSCs during *in vitro* expansion

**Fig. 1:**
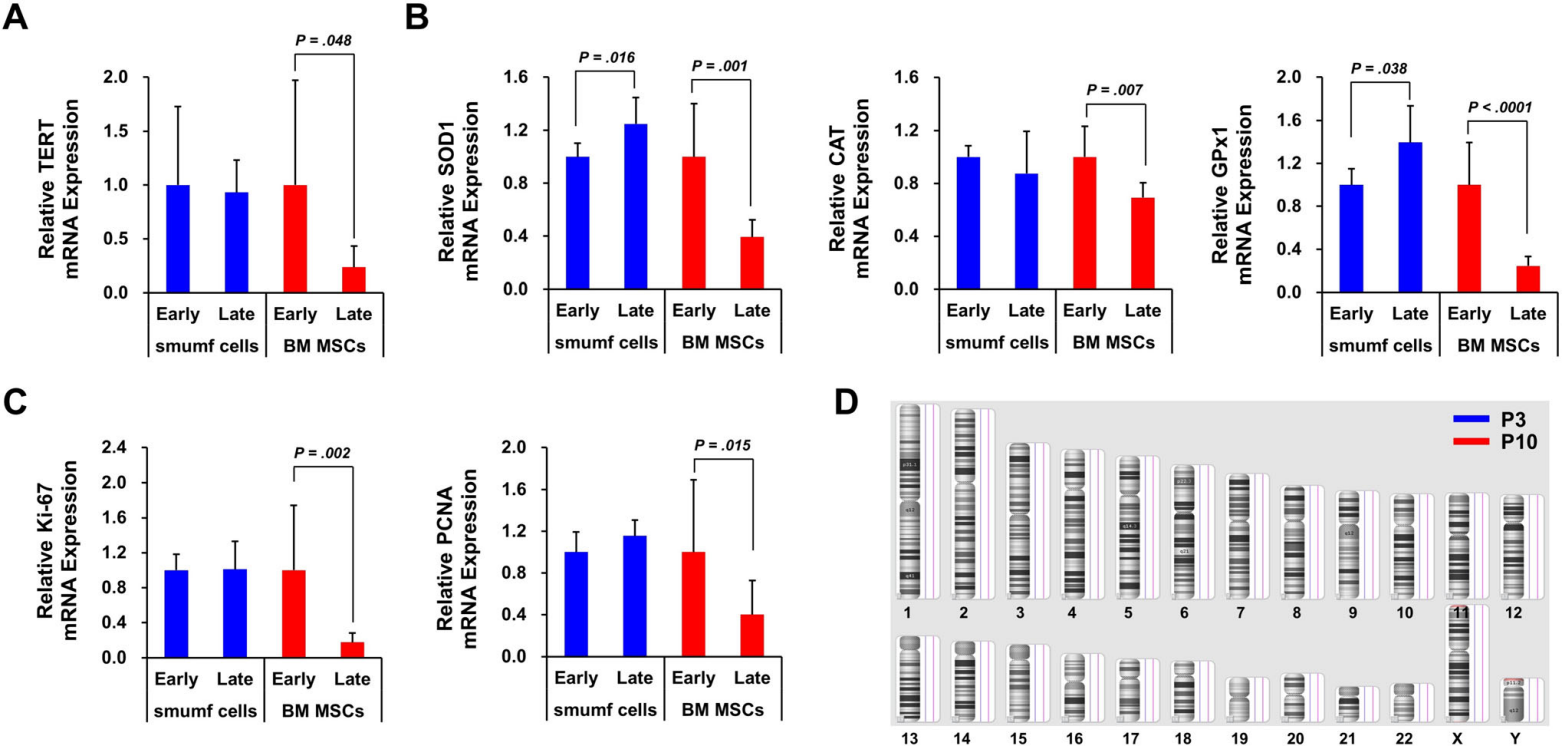
Alterations in aging hallmarks in smumf cells and BM MSCs during *in vitro* expansion. **A–C** The mRNA expression levels related to telomere attrition (TERT), antioxidants (SOD1, CAT, and GPx1), and proliferation (Ki-67 and PCNA) in early (Early) and late-passage (Late) of smumf cells and BM MSCs were evaluated using RT-qPCR. For late-passage, P10 was used for smumf cells, while P6–P10 were used for BM MSCs. **D** Analysis of genomic stability using a CNV array. Genomic representation demonstrates no CNVs in smumf cells at P3 and P10. The blue line represents P3, while the red line represents P10. All data are presented as mean ± standard deviation

During long-term culture *in vitro*, MSCs can undergo senescence and gradually display typical morphological features [22]. To assess the impact of *in vitro* expansion on MSCs, we analyzed the mRNA expression associated with the aging process. Serial passaging significantly decreased mRNA expression of telomerase reverse transcriptase (TERT) by 76.47% in BM MSCs, but smumf cells showed no significant changes (Fig. 1A). Regarding antioxidants, BM MSCs from late-passage (Late) exhibited a significant decrease in mRNA expression of superoxide dismutase type 1 (SOD1), catalase (CAT), and glutathione peroxidase 1 (GPx1) by 62.13%, 31.56%, and 75.64%, respectively, compared to those from early-passage (Early). Conversely, smumf cells showed a significant increase in mRNA expression of SOD1 and GPx1 by 1.25-fold and 1.40-fold, respectively (Fig. 1B). Proliferation-related genes, kiel-67 (Ki-67) and proliferating cell nuclear antigen (PCNA), showed no changes in expression in smumf cells regardless of passage. However, late-passage BM MSCs showed significant reductions of 82.37% in Ki-67 and 59.92% in PCNA mRNA expression, respectively, compared to those from early-passage (Fig. 1C). No CNVs were observed between smumf cells from P3 to P10, indicating sustained genomic stability following serial passaging (Fig. 1D). These results collectively demonstrate that while BM MSCs displayed hallmarks of aging, smumf cells maintained their phenotypic characteristics resiliently against these aging markers. Consequently, P10, representing the late-passage of smumf cells, was selected for subsequent experiments.

### Growth factors secretion by late-passage smumf cells under an IL-1*β*-induced tendinopathic environment

**Fig. 2:**
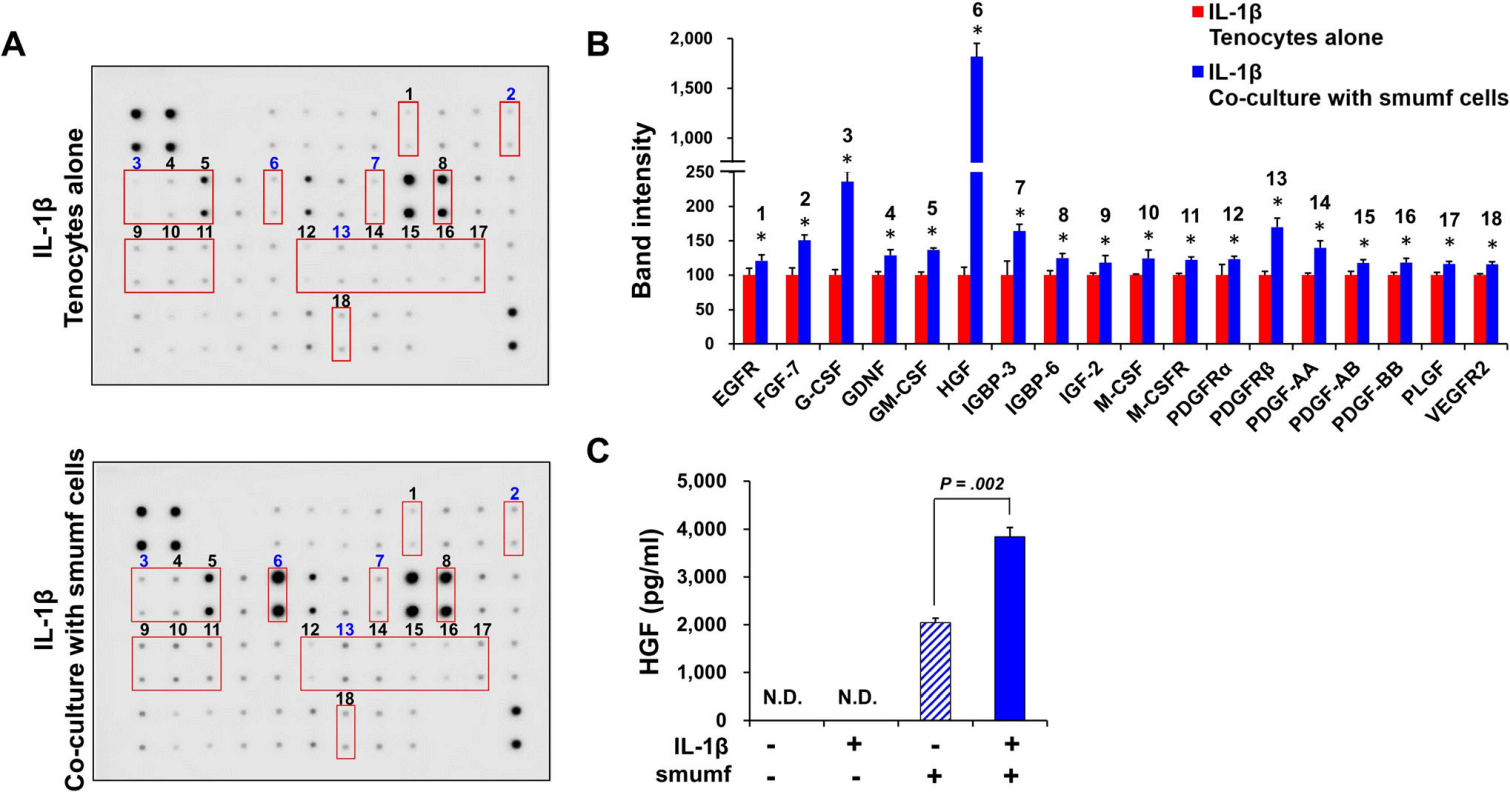
Growth factors secretion by late-passage smumf cells under an IL-1*β*-induced tendinopathic environment. **A** The human growth factor array was performed on medium cultured for 2 days in IL-1*β* treatment. Multiplex array membrane images of tenocytes (top) and tenocytes co-cultured with smumf cells (bottom). At least a 1.20-fold significant increase in GFs in smumf cells co-culture compared to tenocytes alone was indicated by a red box, and a greater than 1.50-fold increase in GFs was highlighted by blue letters. **B** Band intensity was quantified using image J. **C** The concentration of HGF was measured using ELISA. All data are presented as the mean ± standard deviation. *p < 0.05 indicates significant differences when compared with tenocytes alone. “N.D.” means “not detected”

IL-1*β*, a prominent pro-inflammatory cytokine frequently present in tendon disorders, was employed to simulate the tendinopathy environment [27, 28]. To identify the profile of various types of GFs secreted by smumf cells co-culture at P10 under an IL-1*β*-induced tendinopathy environment, we conducted a human growth factor array analysis using co-cultured supernatant. In an inflammatory environment, 18 out of the 41 GFs exhibited at least a 1.20-fold significant increase in smumf cells co-culture compared to tenocytes alone. In particular, GFs secreted with more than a 1.50-fold increase included hepatocyte growth factor (HGF) at 18.19-fold, granulocyte colony-stimulating factor (G-CSF) at 2.36-fold, platelet-derived growth factor receptor *β* (PDGFR-*β*) at 1.70-fold, insulin-like growth factor binding protein 3 (IGBP-3) at 1.64-fold, and fibroblast growth factor-7 (FGF-7) at 1.51-fold (Fig. 2A, B). To further confirm the growth factor array results, we performed ELISA. As a result, HGF secretion reached 2,043 pg/ml in the smumf cells co-culture and exhibited a 1.88-fold increase following IL-1*β* treatment (Fig. 2C). Collectively, these data revealed that smumf cells at P10 secreted significant amounts of diverse GFs, not only in a normal environment but also in an IL-1*β*-induced tendinopathic environment, with HGF being the most prominently secreted.

### Effects of smumf cells on the expression of tenogenic markers in tenocytes under an IL-1*β*-induced tendinopathic environment

**Fig. 3:**
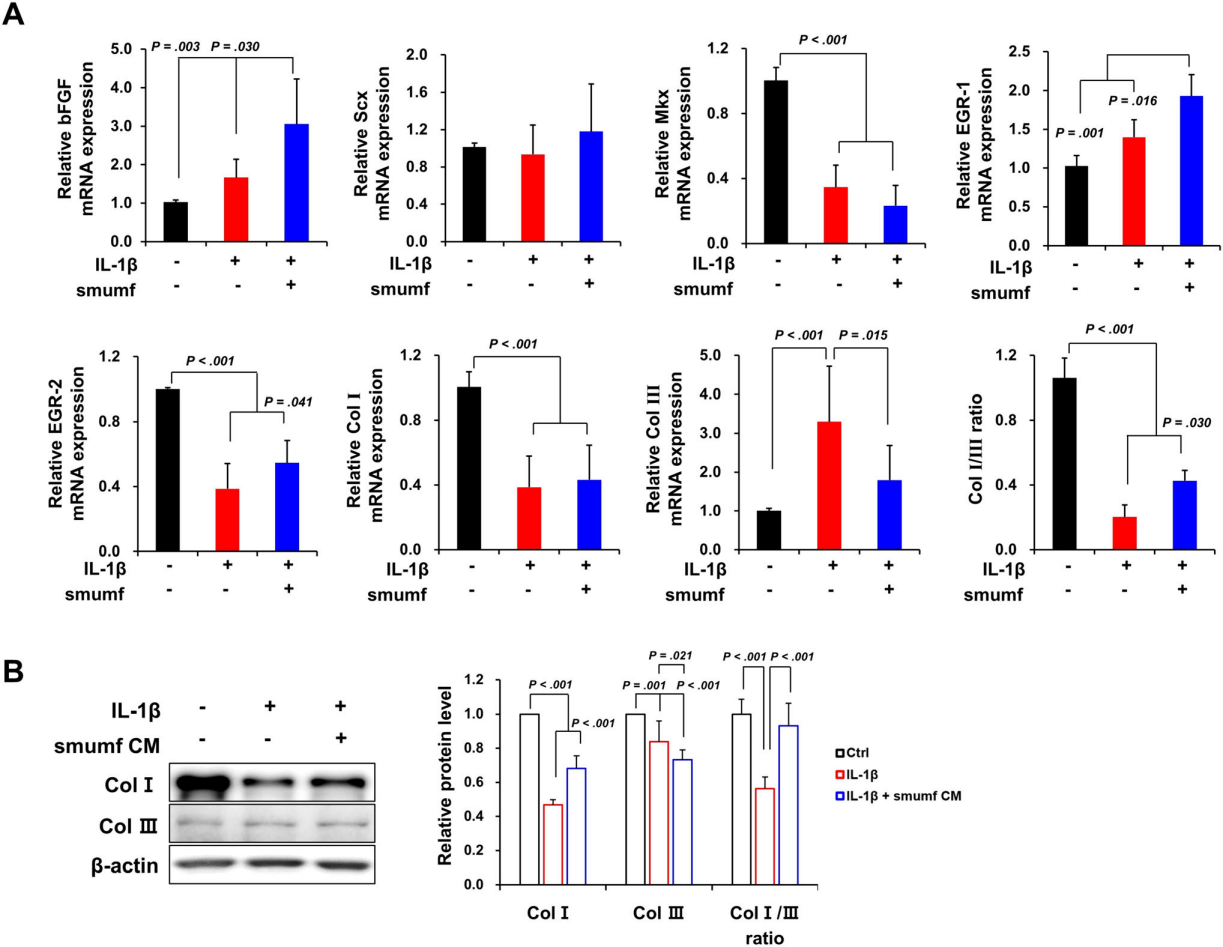
Effects of smumf cells on the expression of tenogenic markers in tenocytes under an IL-1*β*-induced tendinopathic environment. **A** The expression of bFGF, Scx, Mkx, EGR-1, EGR-2, Col I, and Col III in tenocytes was evaluated at the mRNA expression level using RT-qPCR. **B** The protein expression levels of Col I and Col III in tenocytes were analyzed by Western blotting and quantified by densitometry. The Col I/III ratio was calculated by dividing the expression level of Col I by that of Col III. All data are presented as mean ± standard deviation

Using RT-qPCR, we determined changes in the mRNA expression of tendon-relative genes in tenocytes by smumf cells co-culture for 2 days. IL-1*β* treatment resulted in a decrease in mRNA expression of mohawk homeobox (Mkx) by 65.51%, early growth response (EGR)-2 by 61.38%, and collagen type I (Col I) by 61.55% in tenocytes. Conversely, an increase was observed in bFGF by 1.66-fold, EGR-1 by 1.40-fold, and collagen type III (Col III) by 3.30-fold (Fig. 3A). However, smumf cells co-culture significantly increased the mRNA expression of bFGF, EGR-1, and EGR-2 by 1.84-fold, 1.38-fold, and 1.41-fold, respectively, compared to IL-1*β*-induced tenocytes. Additionally, smumf cells co-culture significantly decreased the mRNA expression of Col III by 45.56%, while increasing the Col I/III ratio by 2.11-fold (Fig. 3A). At the protein level, IL-1*β* treatment significantly reduced Col I (53.28%), Col III (16.18%), and the Col I/III ratio (43.66%) in tenocytes. However, smumf cells co-culture significantly increased Col I by 1.46-fold and the Col I/III ratio by 1.66-fold (Fig. 3B). Overall, co-culturing smumf cells at P10 in an inflammatory environment increased the expression of tendon-related genes in tenocytes.

### Effects of smumf cells on the synthesis of inflammatory cytokines, matrix-degradation enzymes, and apoptosis in IL-1*β*-treated tenocytes

**Fig. 4:**
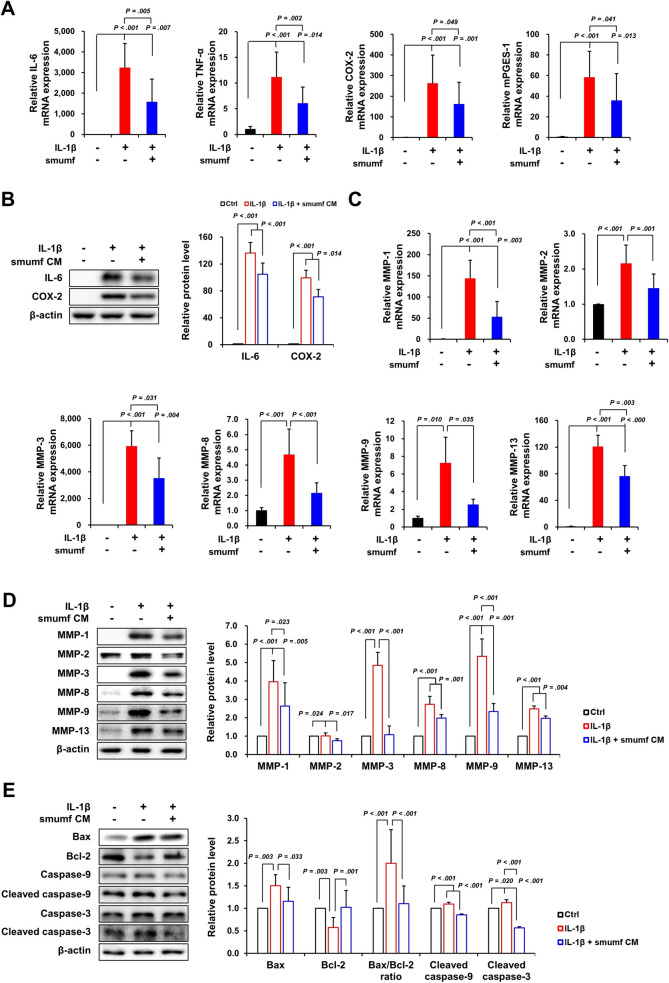
Effects of smumf cells on the synthesis of inflammatory cytokines, matrix-degradation enzymes, and apoptosis in IL-1*β*-treated tenocytes. **A, C** For mRNA expression analysis, tenocytes treated with IL-1*β* for 6 h were then co-cultured with smumf cells for 48 h. **B, D, E** To assess protein expression levels in tenocytes, cells initially treated with IL-1*β* for 6 h were subsequently exposed to smumf CM for 24 h. Band intensity was quantified using image J. All data are presented as the mean ± standard deviation

Inflammation plays a key role in the appearance of tendinopathy, especially in the early phase [29]. To investigate the paracrine effects of smumf cells in regulating inflammatory responses, tenocytes were co-cultured with smumf cells and treated with CM to assess the expression of inflammatory markers via RT-qPCR and Western blot analysis. As shown in Fig. 4A, IL-1*β* treatment resulted in a significant increase in the mRNA expression of IL-6 by 3237.57-fold, TNF-*α* by 11.16-fold, cyclooxygenase-2 (COX-2) by 262.67-fold, and microsomal prostaglandin E synthase-1 (mPGES-1) by 58.47-fold in tenocytes. On the other hand, smumf cells co-culture significantly reduced the expression of IL-6 (51.28%), TNF-*α* (45.64%), COX-2 (38.18%), and mPGES-1 (38.60%) in IL-1*β*-treated tenocytes. Similar to the findings presented in Fig. 4A, IL-1*β* treatment significantly increased the protein expression levels of IL-6 (136.35-fold) and COX-2 (99.38-fold) in tenocytes. Conversely, CM treatment reduced IL-6 and COX-2 protein expression by 23.28% and 28.28%, respectively, in tenocytes (Fig. 4B). We subsequently examined the effect of smumf cells on the expression of matrix-degradation enzymes in IL-1*β*-treated tenocytes using RT-qPCR and Western blot analysis. IL-1*β* treatment upregulated mRNA expression of MMP-1 (144.22-fold), MMP-2 (2.16-fold), MMP-3 (5937.11-fold), MMP-8 (4.69-fold), MMP-9 (7.25-fold), and MMP-13 (120.75-fold) in tenocytes. Conversely, smumf cells co-culture significantly downregulated the mRNA expression of MMP-1 (63.10%), MMP-2 (32.56%), MMP-3 (40.50%), MMP-8 (53.77%), MMP-9 (64.94%), and MMP-13 (36.72%) in IL-1*β*-treated tenocytes, as shown in Fig. 4C. As demonstrated in Fig. 4C, IL-1*β* treatment elevated the protein expression levels of all MMPs in tenocytes. Furthermore, smumf CM treatment significantly reduced the expression of MMP-1 (33.60%), MMP-2 (26.63%), MMP-3 (77.88%), MMP-8 (27.27%), MMP-9 (56.30%), and MMP-13 (20.67%) (Fig. 4D). Next, to evaluate the effect of smumf cells co-culture on IL-1*β*-induced apoptosis, we analyzed the protein expression levels of B-cell lymphoma 2 (Bcl-2), Bcl-2 antagonist X (Bax), Caspase-9, and Caspase-3 in tenocytes using Western blot analysis. Consequently, IL-1*β* treatment of tenocytes resulted in a 2.00-fold increase in the Bax/Bcl-2 ratio, attributable to an increase in Bax and a decrease in Bcl-2, and also elevated the protein expression levels of Cleaved caspase-9 (1.10-fold) and Cleaved caspase-3 (1.13-fold). Conversely, smumf CM treatment led to a significant reduction in the Bax/Bcl-2 ratio (44.85%) and decreased the expression of Cleaved caspase-9 (22.25%) and Cleaved caspase-3 (49.75%) in IL-1*β*-treated tenocytes (Fig. 4E). Taken together, these results indicated that smumf cells at P10 attenuated IL-1*β*-induced synthesis of inflammatory cytokines, matrix-degradation enzymes, and apoptosis in tenocytes.

### Effects of smumf cells on NF-κB and MAPK signaling pathways in IL-1*β*-treated tenocytes

**Fig. 5:**
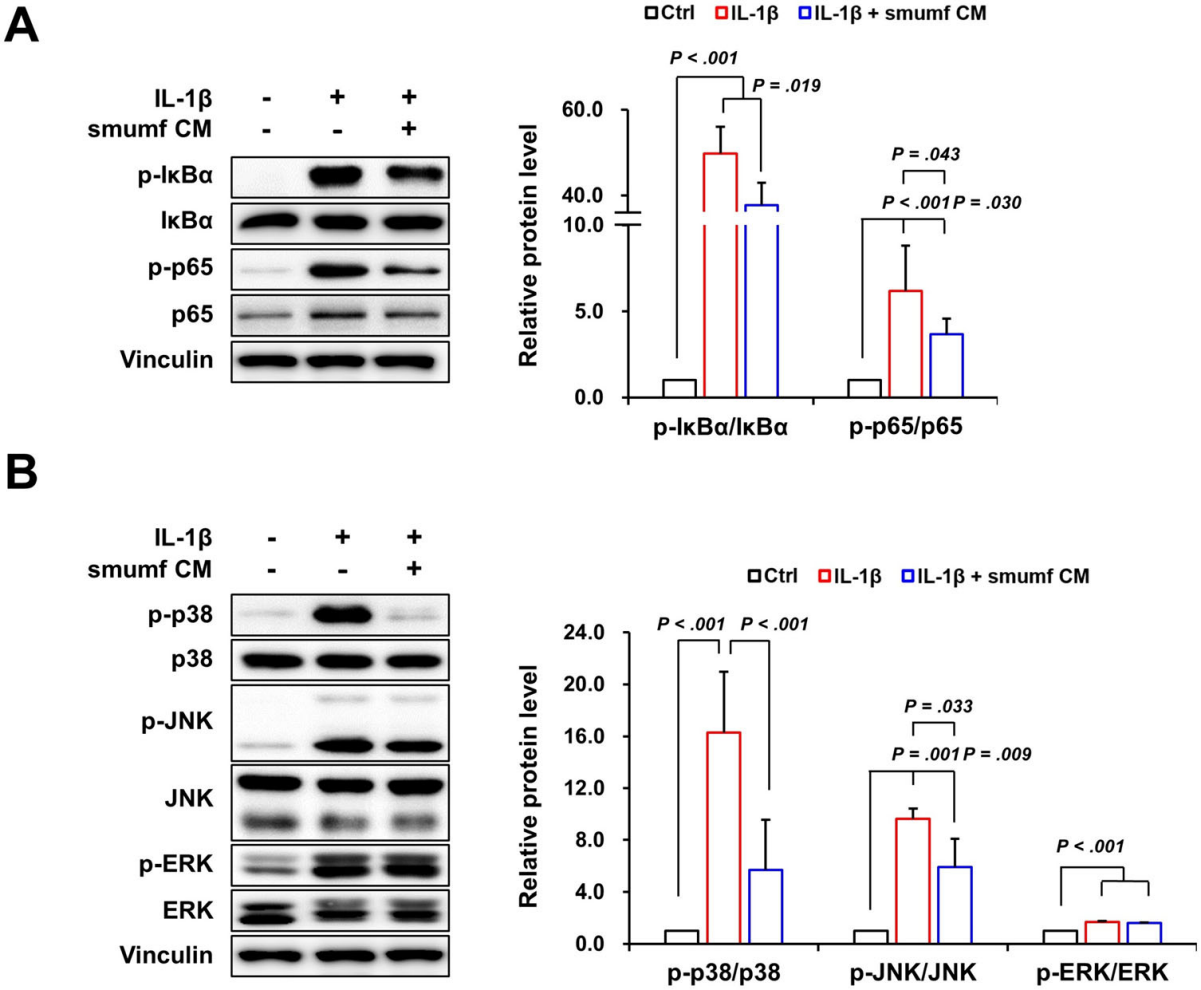
Effects of smumf cells on NF-κB and MAPK signaling pathways in IL-1*β*-treated tenocytes. **A** Tenocytes were treated with 10 ng/ml IL-1*β* and smumf CM for 60 min. The protein expression levels of phosphorylated IκBα, IκBα, phosphorylated p65, and p65 in tenocytes were analyzed by Western blotting and quantified by densitometry. **B** Tenocytes were treated with 1 ng/ml IL-1*β* and smumf CM for 30 min. The protein expression levels of phosphorylated p38, p38, phosphorylated JNK, JNK, phosphorylated ERK, and ERK in tenocytes were analyzed by Western blotting and quantified by densitometry. All data are presented as mean ± standard deviation

To investigate the anti-inflammatory mechanism of smumf cells, we examined the activation of the NF-κB pathway in tenocytes using Western blot analysis. The IL-1*β* treatment induced phosphorylation levels of IkappaB alpha (IκBα) and p65, whereas the smumf CM notably reduced the protein expression levels of phosphorylated IκBα and p65 in tenocytes. This resulted in a 24.16% decrease in the phosphorylated IκBα/IκBα ratio and a 40.56% decrease in the phosphorylated p65/p65 ratio compared to IL-1*β*-treated tenocytes (Fig. 5A). We next analyzed the protein expression levels of p38, jun N-terminal kinase (JNK), and extracellular signal-regulated kinase (ERK) using Western blot analysis to determine whether smumf cells inhibit the activation of the MAPK pathway. IL-1*β* treatment induced the activation of p38, JNK, and ERK through phosphorylation. smumf CM treatment significantly inhibited the phosphorylation of p38 and JNK, leading to a 65.08% reduction in the phosphorylated p38/p38 ratio and a 38.52% reduction in the phosphorylated JNK/JNK ratio, compared to IL-1*β*-treated tenocytes. In contrast, this treatment did not alter the phosphorylated ERK/ERK ratio, as shown in Fig. 5B. Collectively, these results showed that smumf cells at P10 inhibited the NF-κB and MAPK signaling pathway in IL-1*β*-treated tenocytes.

### Effects of smumf cells on tenocyte proliferation in different environments

**Fig. 6:**
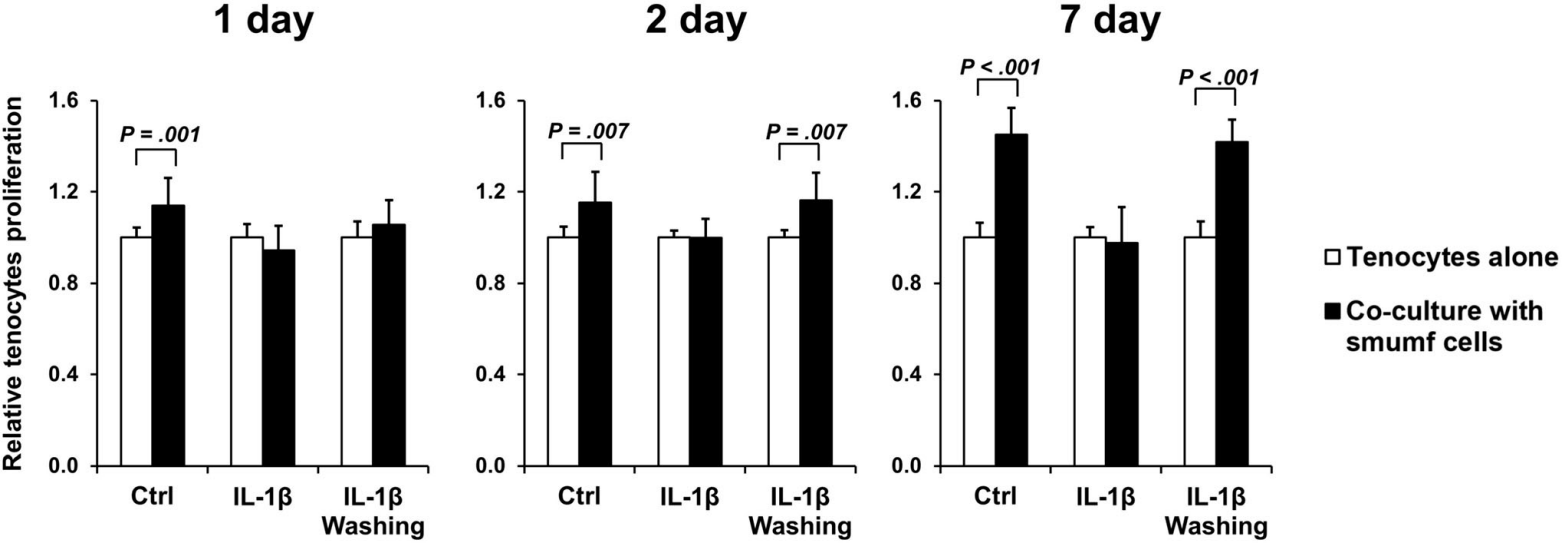
Effects of smumf cells on tenocyte proliferation in different environments. The proliferation of tenocytes was assessed using the WST-1 assay after 1, 2, and 7 days of smumf cells co-culture in various environments. Control (Ctrl): a normal environment; IL-1*β*: an environment with inflammation induced by IL-1*β* treatment; IL-1*β* Washing: an environment where inflammation was removed by washing following 6 h of IL-1*β* treatment. All data are presented as the mean ± standard deviation. *P* values were calculated in comparison with the control tenocytes

To evaluate the effects of smumf cells co-culture at P10 in various environments, tenocyte proliferation was measured using the WST-1 assay after 1, 2, and 7 days of co-culture. The co-culture environments were as follows: (1) a normal environment (Ctrl); (2) an environment in which inflammation was induced by IL-1*β* treatment (IL-1*β*); and (3) an environment from which IL-1*β*-induced inflammation was removed through washing after 6 h (IL-1*β* washing). As shown in Fig. 6, smumf cells co-culture for 1, 2, and 7 days in a normal environment significantly increased the proliferation of tenocytes by 1.14-fold, 1.15-fold, and 1.45-fold, respectively. Conversely, smumf cells co-culture in the presence of IL-1*β* did not affect tenocyte proliferation at any of the time points. Interestingly, it was confirmed that the proliferation of tenocytes significantly increased 1.16-fold and 1.42-fold by smumf cells co-culture for 2 and 7 days in the IL-1*β* washing (Fig. 6). In summary, smumf cells increased tenocyte proliferation only in a non-inflammatory environment.

## Discussion

The most important findings of this study are (1) late-passage smumf cells at P10 did not exhibit aging features, unlike BM MSCs, even when compared to early-passage cells at P3, (2) smumf cells at P10 secreted 2,043 pg/ml of HGF under a normal environment, and this secretion increased by 1.88-fold under an IL-1*β*-induced tendinopathic environment, (3) co-culture of smumf cells with tenocytes in the tendinopathic environment led to a 1.46-fold increase in the protein expression level of Col I in tenocytes, thereby enhancing the Col I/III ratio by 1.66-fold, (4) smumf cells significantly downregualted the expression levels of MMP-1, -2, -3, -8, -9, and -13 in tenocytes under an IL-1*β*-induced tendinopathic environment. Notably, smumf cells at P10 effectively attenuated IL-1*β*-induced activation of the NF-κB and MAPK signaling pathways in tenocytes, and (5) the co-culture of smumf cells in a tendinopathic environment did not affect the proliferative capacity of tenocytes. However, the removal of the tendinopathic environment resulted in a 1.42-fold increase in tenocyte proliferation, which was attributed to the presence of smumf cells in the co-culture. Collectively, these findings suggest that late-passage smumf cells exert anti-inflammatory effects on tenocytes derived from degenerative rotator cuff tears under a tendinopathic environment, primarily through the secretion of GFs.

Real-world clinical applications require substantial numbers of cells, making it essential to cultivate large quantities from a single tissue source. However, serially passaged cells often exhibit signs of aging, including telomere attrition, decreased proliferation, and elevated levels of reactive oxygen species (ROS) [15, 30–32], which ultimately lead to cellular damage [33]. Therefore, it is crucial to identify these changes in late-passage MSCs to ensure the reliable production of large quantities of cells. In the present study, smumf cells at P10, unlike BM MSCs, consistently demonstrated mRNA expression of TERT. Moreover, a notable elevation in the mRNA expression of SOD1 and GPx1 was observed. Some studies have reported a decrease in the mRNA expression of TERT in aged MSCs. However, continuous expression of TERT has been shown to preserve its enzymatic activity and support cell proliferation [19]. The smumf cells not only maintained mRNA expression of TERT but also sustained the expression of proliferation-related genes, including Ki-67 and PCNA. This indicates that these cells retain their proliferative capacity in the absence of telomere attrition. Oxidative stress is a critical factor in the pathogenesis of tendinopathy, and the presence of antioxidants has been demonstrated to exert cytoprotective effects on tenofibroblasts [34]. Consequently, therapeutic strategies aimed at promoting healing and mitigating oxidative stress are advisable. In our study, repeated passaging of BM MSCs resulted in a significant decrease in the mRNA expression of SOD1, CAT, and GPx1. In contrast, smumf cells exhibited a significant increase in the mRNA expression of SOD1 and GPx1. Several studies have reported a reduction in the mRNA expression of SOD1, CAT, and GPx1 in aged BM MSCs [32, 35]. Similarly, Khanh et al. found that adipose tissue-derived MSCs (AD MSCs) from elderly individuals exhibited a significantly higher ROS levels compared to AD MSCs from infants [36]. Given that smumf cells are derived from UC tissue, they are hypothesized to exhibit greater resistance to aging than BM MSCs. CD146 serves as a valuable marker for assessing the aging status of MSCs. Its expression was found to decrease in UCB MSCs with passaging, from 66.0% at P9 to 26.1% at P13 [20]. smumf cells isolated using the MCE method exhibited significantly higher CD146 expression (73.4%) compared to cells isolated by the enzymatic digestion method, and this expression level was sustained until P20 [16]. Previous studies suggested that late-passage MSCs may be more efficacious in disease treatment than early-passage MSCs. For instance, late-passage BM MSCs at P10 exhibited significant improvements in pain behavior and reduced serum TNF-*α* concentrations compared to early-passage BM MSCs following intra-articular injection in an OA model [21]. Similarly, late-passage UC MSCs at P15 exhibited increase levels of the anti-inflammatory mediator heme oxygenase-1 (HMOX-1) and a downregulation of proinflammatory cytokines IL-1*α*, IL-1*β*, and interferon (IFN)-γ, leading to enhanced immunosuppressive activity [23]. Therefore, given that smumf cells at P10, a late-passage, retain their functional capacity without exhibiting significant signs of aging, they are highly suitable candidates for regenerative medicine applications requiring large quantities of cells, such as tendinopathy treatment.

MSCs possess a crucial capacity for tissue regeneration and repair, largely due to their ability to secrete a diverse range of paracrine factors [37]. Among MSCs derived from various tissues, including periodontal ligaments, AD, and UC, UC MSCs are particularly noted for their high secretion levels of HGF, with a concentrations reported at 180 pg/ml [38]. In comparison, smumf cells at P10 secrete HGF at levels 11.35-fold higher than those observed in UC MSCs at P5. Given the significant presence of IL-1*β* in tendinopathic environments [27, 39], it is crucial to investigate the secretion of GFs by MSCs under these clinically relevant conditions. In this study, we confirmed that smumf cells at P10 produced HGF at levels 1.88-fold higher in an IL-1*β*-induced tendinopathic environment than in a normal environment. Collectively, these findings suggest that smumf cells at P10 secrete a diverse and substantial quantity of GFs in both normal and IL-1*β*-induced tendinopathic environments.

IL-1*β* treatment is known to diminish tenocyte characteristics by a reducing the expression of key tendon-associated genes, including scleraxis (Scx), EGR-1, and Col I [40]. Our study confirmed that co-culturing smumf cells led to increased expression of bFGF, EGR-1, and EGR-2 mRNA, and Col I protein in IL-1*β*-treated tenocytes. This effect led to a significant increase in the Col I/III ratio, which is used to analyze the collagen composition during different phases of tendon healing [41]. bFGF is recognized as a potent activator of the EGR genes [42]. The EGR genes serve as a pivotal transcriptional regulator intimately associated with cell growth, survival, and apoptosis, and it plays a crucial role in tendon development and repair [43–46]. The introduction of the bFGF gene via an adeno-associated viral type-2 (AAV2) vector into severed tendons has been shown to increase the expression of both Col I and Col III [47, 48]. Moreover, the transplantation of tendon stem cells (TSCs) overexpressing EGR-1 significantly improved recovery outcomes in a murine Achilles tendon injury model [49]. Our findings suggest that in an IL-1*β*-induced tendinopathic environment, smumf cells co-culture upregulates the expression of EGR genes and Col 1 by inducing bFGF expression in tenocytes. Therefore, smumf cells are considered reasonably safe for clinical application in tendon repair and regeneration, as they improve collagen composition without the need for gene modification.

Tendinopathy is a chronic tendon disorder characterized by both degeneration and inflammation [50]. Particularly, accumulated inflammation induces ECM degradation, thereby disrupting tissue homeostasis and affecting cell survival [51]. The typical matrix-degrading enzymes are as follows: collagenases (MMP-1, -8, and -13), which break down native Col I-III; gelatinases (MMP-2 and -9), responsible for the cleavage of denatured collagens; and stromelysins (MMP-3 and -10), involved in the degradation of proteoglycans, fibronectin, and Col III-V [52]. IL-1*β* treatment increases the expression of MMP-1, -2, -3, -8, -9, and -13 in equine tenocytes [53]. However, research on the potential of MSCs to mitigate the IL-1*β*-induced elevated expression of MMPs in human tenocytes is limited. Therefore, it is crucial to detect alterations in the expression of various MMPs within the IL-1*β*-induced tendinopathic environment of human tenocytes. Previous studies have shown that co-culturing AD MSCs in the presence of IL-1*β* did not alter the mRNA expression of MMP-1 and MMP-3 in tenocytes [54]. In contrast, our study found that smumf cells co-culture significantly decreased the expression of MMP-1, -2, -3, -8, -9, and -13 in IL-1*β*-treated tenocytes, with the most pronounced reductions observed in MMP-1, -3, -8, and -9. These findings suggest that smumf cells can effectively alleviate ECM degradation of these collagen types in tenocytes under an IL-1*β*-induced tendinopathic environment. In tendinopathy, MAPK pathway regulate various physiological and biochemical activities, including the secretion of inflammatory factors, synthesis and degradation of ECM, and apoptosis [55]. Specifically, ERK is involved in the regulation of cell proliferation, while p38 and JNK mediate apoptotic and inflammatory responses [56, 57]. In this study, we found that co-culturing smumf cells significantly reduced the phosphorylation of p38 and JNK, which may be associated with the decreased expression of pro-inflammatory markers such as IL-6, TNF-α, COX-2, and mPGES-1, as well as reduced MMP synthesis and apoptosis in IL-1*β*-treated tenocytes. The NF-κB pathway is involved in all stages of tendon healing and has been shown to activate the MAPK pathway [57, 58]. Tectorigenin, derived from *Belamcanda chinensis*, has been reported to prevent tendinopathy by inhibiting the MAPK and NF-κB pathways in TNF-*α*-treated TSCs [57]. Most studies have focused on the therapeutic effects of early-passage MSCs on tendinopathy [59, 60]. However, research on the inhibition of the NF-κB and MAPK pathways by late-passage MSCs in the context of tendinopathy is limited. In this study, we observed that co-culturing smumf cells resulted in reduced phosphorylation of IκBα and p65 in the NF-κB pathway, as well as decreased activation of the MAPK pathway in IL-1*β*-treated tenocytes. Several studies have shown that HGF suppresses pro-inflammatory processes in a dose-dependent manner by inhibiting the NF-κB pathway and reducing the expression of COX-2 and mPGES-1 in IL-1*β*-treated tenocytes [61–63]. HGF treatment has also demonstrated maximal effectiveness in restoring tendon fiber alignment and biomechanical properties [64]. Collectively, our findings demonstrate that smumf cells at P10, which secrete HGF, can effectively manage tendinopathy by inhibiting the NF-κB and MAPK signaling pathways in tenocytes, thereby reducing inflammation and highlighting their potential as a cell therapy.

The tendon repair process is characterized by three overlapping phases: inflammation, regeneration, and remodeling [56]. During the regeneration phase, the migration of tenocytes to the injured tissue and their proliferation are crucial for successful tendon healing [65]. Numerous studies have demonstrated the ability of MSCs to significantly enhance tenocyte proliferation [25, 56, 66]; however, Kim et al. reported that UC MSCs at P5 did not affect tenocyte proliferation [60]. It is important to note that these controversial results may not adequately reflect conditions within an inflammatory environment. MSCs exhibit a low survival rate in the presence of inflammatory agents in individuals with OA [67, 68]. Fan et al. identified that inflammatory synovial fluid (SF) in OA contains a variety of inflammatory factors that could diminish the effectiveness of MSC-based treatments, while the removal of these factors enhances chondrocyte migration and proliferation when co-cultured with UC MSCs [69]. Despite these insights, research on the modulation of the inflammatory environment in tendinopathy and its impact on MSC interventions remains limited. In present study, we found that tenocyte proliferation was influenced not only by smumf cells co-culture but also by the presence of an IL-1*β*-induced tendinopathic environment. These results suggest that effectively managing the inflammatory environment may be crucial to amplify the therapeutic of injected smumf cells.

This study had several limitations. First, tenocytes were obtained from degenerative tissues due to the difficulty in acquiring tissue samples from healthy individuals. Consequently, the control group in this study may not fully represent normal physiological conditions of healthy tendons. Second, although *in vitro* models of tendinopathy using IL-1*β* treatment are widely adopted, their ability to accurately reproduce clinical tendinopathy remains questionable. Third, the enhanced therapeutic effect of smumf cells in an environment where inflammatory factors were removed was not confirmed.

In conclusion, this study demonstrates that late-passage smumf cells exert anti-inflammatory effects on tenocytes derived from degenerative rotator cuff tears under a tendinopathic environment, primarily through the secretion of GFs. These findings suggest that late-passage smumf cells could be an effective cell therapy for the management of tendinopathy.

## Supplementary Information

Below is the link to the electronic supplementary material.

Supplementary file1 (DOCX 45 KB)

## Data Availability

The datasets used and/or analyzed during the current study are available from the corresponding author on reasonable request.
