# Supplementary material for: Effects of Late-Passage Small Umbilical Cord–Derived Fast Proliferating Cells on Tenocytes from Degenerative Rotator Cuff Tears under an Interleukin 1β-Induced Tendinopathic Environment
Source: Tissue Eng Regen Med. 2024 Nov 5;21(8):1217–31. doi: 10.1007/s13770-024-00673-x (PMC11589062; doi:10.1007/s13770-024-00673-x)
Supplement: Supplementary file 1 — Supplementary file1 (DOCX 45 KB) [file 13770_2024_673_MOESM1_ESM.docx]

**Supplementary Table S1.** Primer sequences used for RT-qPCR.

| **Gene** | **Sequence (5' - 3')** |
| --- | --- |
| Telomerase reverse transcriptase (TERT) | F: GCCGATTGTGAACATGGACTACG |
|  | R: GCTCGTAGTTGAGCACGCTGAA |
| Kiel-67 (Ki-67) | F: AAGCCCTCCAGCTCCTAGTC |
|  | R: GCAGGTTGCCACTCTTTCTC |
| Proliferating cell nuclear antigen  (PCNA) | F: GGCGTGAACCTCACCAGTAT |
|  | R: TTCTCCTGGTTTGGTGCTTC |
| Superoxide dismutase type 1  (SOD1) | F: CTGAAGGCCTGCATGGATTC |
|  | R: CCAAGTCTCCAACATGCCTCTC |
| Catalase (CAT) | F: AGGGGCCTTTGGCTACTTTG |
|  | R: ACCCGATTCTCCAGCAACAG |
| Glutathione peroxide 1  (GPx1) | F: CCGGGACTACACCCAGATGA |
|  | R: CGTTCTCCTGATGCCCAAAC |
| Glyceraldehyde-3-phosphate dehydrogenase (GAPDH) | F: GTCTCCTCTGACTTCAACAGCG |
|  | R: ACCACCCTGTTGCTGTAGCCAA |

**Supplementary Table S2.** Taqman probes used for RT-qPCR.

| **Gene** | | **Taqman Gene Expression Assay Kit** |
| --- | --- | --- |
| Basic fibroblast growth factor (bFGF) | Hs00266645_m1 | |
| Scleraxis (Scx) | Hs03054634_g1 | |
| Mohawk Homeobox (Mkx) | Hs00543190_m1 | |
| Early growth response-1 (EGR-1) | Hs00152928_m1 | |
| Early growth response-2 (EGR-2) | Hs00166165_m1 | |
| Collagen Type I (Col Ⅰ) | Hs00164004_m1 | |
| Collagen Type III (Col Ⅲ) | Hs00943784_g1 | |
| Cyclooxygenase-2 (COX-2) | Hs00153133_m1 | |
| Interleukin-6 (IL-6) | Hs99999032_m1 | |
| Tumor necrosis factor-alpha (TNF-α) | Hs99999043_m1 | |
| Microsomal prostaglandin E synthase-1 (mPGES-1) | Hs00610420_m1 | |
| Matrix metalloproteinases (MMP)-1 | Hs00899658_m1 | |
| MMP-2 | Hs01548727_m1 | |
| MMP-3 | Hs00968308_m1 | |
| MMP-8 | Hs00233972_m1 | |
| MMP-9 | Hs00957555_m1 | |
| MMP-13 | Hs00233992_m1 | |
| GAPDH | Hs99999905_m1 | |

**Supplementary Table S3.** List of antibodies for Western blotting.

| **Antibody** | **Company (Cat No.)** | **Dilution** |
| --- | --- | --- |
| Col Ⅰ | LSBio (LS-C343921) | 1:1000 |
| Col Ⅲ | Santa Cruz Biotechnology (sc-271249) | 1:500 |
| COX-2 | Cell Signaling Technology (12282) | 1:1000 |
| IL-6 | Cell Signaling Technology (12153) | 1:1000 |
| MMP-1 | Cell Signaling Technology (54376) | 1:500 |
| MMP-2 | Cell Signaling Technology (4022) | 1:500 |
| MMP-3 | Cell Signaling Technology (14351) | 1:500 |
| MMP-8 | Cell Signaling Technology (78399) | 1:500 |
| MMP-9 | Cell Signaling Technology (3852) | 1:500 |
| MMP-13 | Cell Signaling Technology (69926) | 1:500 |
| IkappaB alpha (IκBα) | Cell Signaling Technology (4814) | 1:1000 |
| Phospho- IκBα (p-IκBα) | Cell Signaling Technology (2859) | 1:1000 |
| Nuclear factor kappa-light-chain-enhancer of activated B cells (NF-κB) p65 | Cell Signaling Technology (8242) | 1:1000 |
| Phospho-p65 (p-p65) | Cell Signaling Technology (3033) | 1:1000 |
| p44/42 mitogen activated protein kinase (MAPK) (extracellular-signal-regulated kinase (ERK) 1/2) | Cell Signaling Technology (4695) | 1:1000 |
| Phospho-p44/42 MAPK (ERK1/2) | Cell Signaling Technology (4370) | 1:1000 |
| Phospho-Stress-activated protein kinases (SAPK)/Jun N-terminal kinases (JNK) | Cell Signaling Technology (9252) | 1:1000 |
| Phospho- JNK (p-JNK) | Cell Signaling Technology (9251) | 1:1000 |
| p38 MAPK | Cell Signaling Technology (8690) | 1:1000 |
| Phospho-p38 MAPK (p-p38) | Cell Signaling Technology (9211) | 1:1000 |
| Bcl-2 antagonist X (Bax) | Proteintech (50599-2-Ig) | 1:2000 |
| B-cell lymphoma 2 (Bcl-2) | Proteintech (12789-1-AP) | 1:2000 |
| Caspase-9 | Cell Signaling Technology (9502) | 1:1000 |
| Caspase-3 | Cell Signaling Technology (9662) | 1:1000 |
| β-actin | Santa Cruz Biotechnology (sc-47778) | 1:4000 |
| Vinculin | Cell Signaling Technology (13901) | 1:5000 |
| Anti-rabbit IgG | Cell Signaling Technology (7074) | 1:4000 |
| Anti-mouse IgG | Cell Signaling Technology (7076) | 1:4000 |
